# Histological Characterisation of Small Renal Masses and Incidence of Silent Renal Masses

**DOI:** 10.1155/2008/758073

**Published:** 2008-11-04

**Authors:** Sergio Almenar Medina, Ana Calatrava Fons

**Affiliations:** Department of Pathology, Instituto Valenciano de Oncología, 46009 Valencia, Spain

## Abstract

With the introduction of sonographic and CT examinations, the number of small renal masses detected has increased. Benign neoplastic lesions are usually smaller than 4 cm in size, whilst the most common types of renal cell carcinomas have a mean size greater than that, but we must not forget that a significant number of small masses are renal cell carcinomas; even though the rate of benign cases increases as the diameter of the lesions decreases, therefore, size itself cannot be used to rule out a diagnostic of malignancy and often image characteristics are not enough to predict the nature of the lesion with certainty. In this case, histological confirmation must be recommended. Ideally, the histological study must be conducted on the surgical specimen, even though biopsy can be an option in selected cases.

## 1. INTRODUCTION

Since the beginning of the eighties, with the introduction of sonographic and CT examinations, the number of small renal masses detected is greater than it was previously, when they were discovered by clinical methods [1]. According to a study conducted by The New York University Medical Center, the number of renal masses smaller than 3 cm which were detected in a period of 5 years during the eighties was five times greater than that found in a similar period during the seventies [2] due to, according to Bosniak, the increase in the number of abdominal image studies carried out and to the systematic inclusion of kidneys in these studies. In the Wunderlich series [3], the percentage of tumours of less than 4 cm increased from 28% in 1985 to 61% in 1995.

These lesions can be sporadic or associated to hereditary syndromes, chronic renal failure, or renal transplantation. In the first case, they can be detected during the course of abdominal studies due to renal symptoms or other causes. In the second case, they are detected during the illnesses' follow-up or as a consequence of specific screening programmes.

In every case, masses can be solitary or multiple and may be solid, cystic, or solid-cystic. Depending on their cystic component in images, they can be classified into four categories. Lesions belonging to Bosniak I y II are benign, whereas 59% of Bosniak III and 100% of Bosniak IV are malignant [4].

## 2. CYSTIC RENAL LESIONS

Renal cysts are frequent lesions of variable size, which appear associated to different clinical situations. Histologically, they are lined by a single layer of cells which may be cubical in the beginning but become more or less flattened as the cyst increases in size. However, sometimes this epithelium may develop hyperplastic lesions, giving way to the big discussion that exists about possible malignant transformation.

Simple renal cysts, whether solitary or multiple, are very variable in size but are frequently smaller than 4 cm. Usually, they are autopsy incidental findings and have no clinical relevance.

In polycystic kidney disease (dominant or recessive), cysts have a cuboidal or flattened lining which may proliferate to form papillary structures inside.

In acquired renal cystic disease, which is associated to dialysis, the majority of the cysts measure between 0.5 and 2 cm, but may develop renal cell carcinomas mainly of papillary type. The risk of developing a carcinoma in patients which are undergoing dialysis is greater in those who have developed acquired renal cystic disease [5].

## 3. SOLID AND COMPLEX RENAL MASSES

Solid and complex renal masses are mainly of neoplastic origin but some inflammatory lesions may also have equivocal sonographic images, as it may be seen in Lebret's series, where 22 out of 106 studied lesions turned out to be inflammatory tissues, abscesses, or granulomatous pyelonephritis [6]. On the other hand, most renal cell carcinomas are solid, but 40% of them have a cystic component [7].

Some of the benign neoplastic lesions are usually smaller than 4 cm in size, but we must not forget that, to reach its final size, every lesion must go through this initial stage. Therefore, size itself is not a criterion which can be used to rule out a diagnostic of malignancy.

The most frequently detected benign neoplasms are oncocytomas and angiomyolipomas.

Papillary adenomas are tumours with papillary or tubular architecture of low nuclear grade and 5 mm in diameter or smaller [8]. These are the most common neoplasms of the epithelium of the renal tubules and have been found in 40% of autopsies of patients older than 70 years. Most papillary adenomas are silent, solitary, and occur just below the renal capsule. Histologically, they have tubular, papillary, or tubulopapillary architectures corresponding closely to types 1 and 2 of papillary renal cell carcinoma. Loss of the Y chromosome and a combined trisomy of chromosomes 7 and 17 are the first genetic alterations we can find in papillary tumours and the sole karyotype change in papillary tumours from 2 mm to 5 mm in diameter, all with nuclear grade 1. However, it is not possible to distinguish adenomas and carcinomas by genetic changes, because many carcinomas show only a few genetic alterations [9]. Therefore, the difference between low-grade papillary renal cell carcinoma and adenoma depends mainly on size [8].

Metanephric adenoma is another solid, less frequent, typically benign renal tumour, which ranges widely in size. Jones et al. have reported 7 incidental cases, all of them are less than 1 cm, although symptomatic cases are usually larger than 3 cm [10].

The most common types of renal cell carcinomas have a mean size which is greater than 4 cm, but some unusual types have a mean size of less than 4 cm. According to Nassir, the mean size of multilocular cystic renal cell carcinoma is 3.4 cm [11] and the acquired cystic disease-associated RCC usually has a mean size of around 3 cm and shows peculiar morphological and immunohistochemical features [12, 13]. Normally, they have a microcystic architecture and Fuhrman grade 3. They also describe another group which they refer to as “clear-cell papillary RCC of the end-stage kidneys.”

Papillary renal tumours with oncocytic cells of the adult have, according to Lefevre, a mean size of 3.3 cm, they are intrarenal, with sharp edges and all, except one, have Fuhrman grade 2 [14].

Carcinomas belonging to the hybrid oncocytic tumour variety (which frequently occur in the Birt-Hogg-Dubé syndrome) are usually of a small size and their behaviour is between the oncocytoma and the well-differentiated chromophobe renal cell carcinoma [15].

Finally, 10 out of the 13 tubulocystic renal cell carcinomas described by Yang et al. [16] measured less than 3 cm.

## 4. RELATIONSHIP BETWEEN TUMOURAL SIZE AND HISTOLOGICAL TYPE

One of the main problems when it comes to analysing this relationship is that most of the small renal tumour series are based on clinical and radiological data but histological confirmation lacks [27], especially when lesions are diagnosed incidentally, because the biopsy is not indicated as a routine method [19, 22, 28]. Moreover, the series which includes a histological study uses different criteria to indicate surgery or biopsy, different cutoff points for small masses, and even different pathologic classifications, which make it even more complicated to draw general conclusions (Table 1) [5, 6, 17–25].

Nevertheless, it is clear that a significant number of small solid and complex masses are renal cell carcinomas and they are, according to some authors, more frequent than benign lesions [23, 26], even though the rate of benign cases increases as the diameter of the lesions decreases.

In these cases, it is not possible to predict the behaviour that the lesions will have later neither by their image characteristics [29] nor by their growing speed throughout a short period of time, due to the fact that this speed is not related to the tumoural volume or to the histological grade at a given time [18, 30]. This speed may vary throughout time for a same tumour [31] and can be temporarily zero, even though it is a carcinoma [22]. According to Kunkle, there are no any significant differences between tumours with growth zero during a period of one year and those which have positive growth during the same period at the time of the diagnosis. In both cases, the mean size is of 2 cm and 80% of the lesions of growth zero happened to be carcinomas [17].

According to a recent study by Tabibi [32], amongst renal cell carcinomas, there is no significant relationship between size and histological subtype, even though it is true that, in long series, the size of tumours of the same type tends to gather around a certain value. The usual histological subtypes of renal cell carcinoma have a mean size of more than 4 cm [33] and the same happens with translocation Xp11 renal cell carcinomas [34] and with translocation t(6;11)(p21;q12) [35], where the mean diameter is 6.8 cm and 6.28 cm, respectively. When carcinomas are analysed according to size, it can be observed that when the mean tumoural diameter increases, the ratio of papillary carcinomas decreases and that of chromophobe carcinomas increases (Table 2) [20, 23, 26].

## 5. RELATIONSHIP BETWEEN SIZE AND AGGRESSIVENESS OF RENAL CELL CARCINOMAS

It cannot be categorically assured that the size of a tumour is directly related to its histological grade and its clinical aggressiveness. For example, low-grade tubular-mucinous renal neoplasia (also known as mucinous tubular and spindle cell carcinoma) is a neoplasia with a low grade of aggressiveness and, nevertheless, usually has a mean diameter which is larger than 4 cm [12, 41, 42]. In addition, chromophobe carcinomas are usually larger but less aggressive than clear cell renal cell carcinomas [33]. In the same way, among small renal cell carcinomas, the clear cell subtype is much more frequent than chromophobe carcinoma (Table 3) [5, 6, 17–20, 23, 25, 26, 31, 36–38].

Amongst small size tumours, there is a higher rate of low-grade lesions and this percentage tends to decrease as the tumoural size increases (Table 4) [23, 26, 39], but we must not forget that among carcinomas which are smaller than 4 cm, there is a significant ratio, between 6% and 50%, of high-grade tumours (Table 5) [5, 6, 18, 20, 21, 23, 25, 39, 40].

In Schlomer's series [26], 16.7% of the tumours which are smaller than 1 cm are high-grade tumours and 38% of the 50 carcinomas with a size equal to or less than 3 cm included in Hsu's series [39] extend beyond the renal capsule.

This last series also shows that there is no significant difference in grade or stage between tumours of less than 3 cm and those of 3 to 5 cm, but these differences do exist between tumours of less than 5 cm and those which are greater. In the results reported by Tabibi [32], extracapsular spread is rare in tumours of less than 4 cm, but he does not find statistically significant differences in grade when the cutoff point is established at 4 cm. Schlomer and Miyagawa's results [26, 43] point towards this same direction. This is why some authors question that the cutoff point is established at 4 and not at 5 cm [39, 43].

## 6. INCIDENTAL RENAL TUMOURS

Most of the incidentally diagnosed lesions are benign. In a classical series which studied 205 incidental lesions of less than 1 cm found in autopsies, the most common was medullary fibrous nodules (159), followed by cortical adenoma (49), leiomyoma (12), lipoma (7), and myolipoma (13) [44].

Renal carcinomas which are incidentally diagnosed represent between 15 and 60% of the total number of carcinomas, depending on the series.

A lot of them are smaller than 4 cm [28]. Generally speaking, carcinomas discovered incidentally are smaller than those which are symptomatic [45, 46]. Their mean diameter is 5.7 cm in contrast with the 8.7 cm in symptomatic cases. Moreover, the mean size has reduced notably thanks to image techniques. The mean diameter of renal tumours incidentally found in autopsies at the University of Iowa decreased from 4.63 cm in the fifties to 1.65 cm in the nineties [20]. They are also associated with a lower stage and a lower nuclear grade [47], as well as with the increasing age of patients [48].

Therefore, it would be reasonable to consider incidental carcinomas as a group with its own clinical and pathological significance, even though we cannot establish at present whether they are discovered incidentally because they still small or because they have their own particular biological characteristics which make them behave in a less-aggressive way.

On the other hand, the presence of certain syndromes may influence the size that some tumours reach, as it happens to angiomyolipomas which have a greater mean diameter in the context of tuberous sclerosis than when they appear sporadically [49].

## 7. CONCLUSIONS

A significant rate of small renal masses, discovered either symptomatic or incidentally, are carcinomas. Moreover, up to 50% of carcinomas measuring less than 4 cm are high-grade lesions and some of them extend beyond the renal capsule despite the fact that they have got such a small diameter. Therefore, when a small renal mass is detected, the size itself is not a reliable feature to rule out a diagnostic of malignancy. Unfortunately, there are many times when image characteristics or speed of growth along a period of several months are not enough to predict the nature of the lesion with certainty. In these cases, histological confirmation must be recommended.

Most of the times, the histological study is conducted on the surgical specimen, though biopsy can be considered a good option if surgery represents a high risk for the patient. The problem is that the smaller a mass is, the more difficult it is to get the right sample; but the bigger it is, the less representative the whole of the lesion is. That is why there is no general agreement about biopsy indications at the moment. On the other hand, surgical resection in the early stage of the tumour is still the best treatment option for renal cell cancer, and small masses are good candidates for conservative techniques. From this point of view, surgery implies a double benefit because it is a good therapeutic choice and provides the most accurate diagnosis.

Risks and benefits must be evaluated in every single case taking into account the particular clinical situation of the patient as well as the available technical means and the expertise of the medical team involved.

## Figures and Tables

**Table 1:** Small renal masses with histological confirmation. *N*: total number of cases; *n*: number of cases; RCC: renal cell carcinoma; OM: other malignant tumors; Onc: oncocytoma; AML: angiomyolipoma; AP: papillary adenoma; OB: other benign lesions.

	Size	*N*	RCC		OM		Onc		AML		AP		OB	
			*n*	%	*n*	%	*n*	%	*n*	%	*n*	%	*n*	%
Kunkle, 2007 [17]	Median 2 cm	42	37	88.1	0	0.0	4	9.5	0	0.0	0	0.0	1	2.4
Lebret, 2007 [6]	Median 3 cm	135	55	40.7	13	9.6	15	11.1	4	2.9	0	0.0	48	35.6
Chawla, 2006 [18]	Most < 4 cm	21	17	80.9	0	0.0	4	19.1	0	0.0	0	0.0	0	0.0
Vasudevan, 2006 [19]	All < 5 cm	70	41	58.6	6	8.6	14	20.0	9	12.9	0	0.0	0	0.0
Neuzillet, 2005 [5]	Mean 3.7 cm	15	12	80.0	0	0.0	1	6.7	0	0.0	1	6.7	1	6.7
Mindrup, 2005 [20]	Mean 1.7 cm	73	28	38.6	2	2.7	1	1.4	4	5.5	22	30.1	16	21.9
Volpe, 2004 [21]	All < 4 cm	9	8	88.9	0	0.0	1	11.1	0	0.0	0	0.0	0	0.0
Wehle, 2004 [22]	All < 4 cm	5	4	80.0	0	0.0	1	20.0	0	0.0	0	0.0	0	0.0
Frank, 2003 [23]	All < 4 cm	2935	2559	87.2	0	0.0	274	9.3	67	2.3	16	0.5	19	0.7
Bosniak, 1995 [24]	All < 3.5 cm	26	22	84.6	0	0.0	4	15.4	0	0.0	0	0.0	0	0.0
Silverman, 1994 [25]	All < 3 cm	35	27	77.1	2	5.7	0	0.0	1	2.9	0	0.0	5	14.3

**Table 2:** Distribution of histological subtypes of renal cell carcinoma depending on size. RCC: renal cell carcinoma; *N*: total number of RCC; *n*: number of each subtype; RCCcc: clear cell RCC; RCCp: papillar RCC; RCCchr: chromophobe RCC.

	RCC	RCCcc		RCCp		RCCchr	
	*N*	*n*	%	*N*	%	*n*	%
Schlomer, 2006 [26]							

0-1 cm	6	5	83,3	0	0,0	1	16,7
1-2 cm	38	25	65,8	10	26,3	3	7,9
2-3 cm	63	49	77,8	12	19,0	2	3,2
3-4 cm	52	43	82,3	9	17,3	0	0,0
4-5 cm	28	22	78,6	4	14,3	1	3,6
> 5 cm	102	81	84,4	11	11,5	4	4,1

Mindrup, 2005 [20]							

Media 1, 7 cm	28	7	35,0	11	68,8	0	0,0
Media 4, 7 cm	40	13	65,0	5	31,2	3	100,0

Frank, 2003 [23]							

0-1 cm			25,6		74,4		0,0
1-2 cm			59,9		38,6		1,5
2-3 cm			70,2		26,0		3,8
3-4 cm			80,2		24,5		3,8

**Table 3:** Percentage of histological subtypes of renal cell carcinoma in small renal masses. RCCcc: clear cell renal cell carcinoma; RCCp: papillary renal cell carcinoma; RCCchr: chromophobe renal cell carcinoma; RCCo: other variants of renal cell carcinoma.

	Size	RCCcc		RCCp		RCCchr		RCCo	
		*n*	%	*N*	%	*n*	%	*n*	%
Kunkle, 2007 [17]	Median 2 cm	24	64,9	12	32,4	0	0,0	1	2,7
Lebret, 2007 [6]	Median 3 cm	41	74,5	10	18,2	4	7,3	0	0,0
Chawla, 2006 [18]	Most < 4 cm	9	52,9	7	41,2	0	0,0	1	5,9
Pankhurst, 2006 [36]	21 of them < 1 mm	2	8,0	22	88,0	0	0,0	1	4,0
Schlomer, 2006 [26]	Only < 4 cm	122	76,7	31	19,5	6	3,8	0	0,0
Vasudevan, 2006 [19]	All < 5 cm	32	78,0	4	9,8	5	12,2	0	0,0
Neuzillet, 2005 [5]	Mean 37 mm	7	58,3	3	25,0	2	16,7	0	0,0
Mindrup, 2005 [20]	Mean 17 mm	7	25,0	11	39,3	0	0,0	10	35,7
Kato, 2004 [31]	All < 4 cm	15	83,3	3	16,7	0	0,0	0	0,0
Frank, 2003 [23]	All < 4 cm	1970	77,0	436	17,0	125	4,9	28	1,1
Shishikura,1996 [37]	All < 2, 5 cm	84	86,6	3	3,1	0	0,0	10	10,3
Silverman, 1994 [25]	All < 3 cm	17	63,0	3	11,1	3	11,1	4	14,8
Yamashita, 1992 [38]	All < 3 cm	26	72,2	0	0,0	7	19,4	3	8,3

**Table 4:** Percentage of low-grade and high-grade carcinomas depending on size. LG: low grade; HG: high grade.

	Schlomer, 2006 [26]		Hsu, 2004 [39]		Frank, 2003 [23]	
	LG %	HG %	LG %	HG %	LG %	HG %
0-1 cm	83,3	16,7	—	—	90,9	9,1
1-2 cm	94,7	5,3	—	—	88,6	11,4
2-3/< 3 cm	71,4	28,6	72,0	28,0	93,6	6,5
3-4 cm	71,1	28,9	—	—	81,3	18,7
4-5/3–5 cm	67,9	32,1	67,8	32,2	77,6	22,4
5-6 cm	53,8	46,2	—	—	69,3	30,7
6-7/> 5 cm	44,4	55,6	40,4	59,6	60,9	39,1
> 7 cm	36,2	63,8	—	—	37,9	62,1

**Table 5:** Distribution of Fuhrman grades in renal cell carcinomas. F1, F2, F3, F4: Furhman grades.

	Size	RCC	F1+F2		F3+F4	
		*n*	*n*	%	*n*	%
Lebret, 2007 [6]	Median 3 cm	57	51	89,5	6	10,5
Chawla, 2006 [18]	Most < 4 cm	17	16	94,1	1	5,9
Neuzillet, 2005 [5]	Mean 37 mm	12	6	50,0	6	50,0
Mindrup, 2005 [20]	Only < 4 cm	159	123	77,4	36	22,6
Peces, 2004 [40]	Only < 4 cm	12	11	91,7	1	8,3
Hsu, 2004 [39]	Only < 3 cm	50	36	72,0	14	28,0
Volpe, 2004 [21]	All < 4 cm	8	4	50,0	4	50,0
Frank, 2003 [23]	Only < 4 cm	480	420	87,5	60	12,5
Silverman, 1994 [25]	All < 3 cm	27	24	88,9	3	11,1
